# Genome Mining-Guided Discovery of the Glycosylated Griseorhodin Congener Ruskamycin

**DOI:** 10.1021/acs.jnatprod.6c00190

**Published:** 2026-05-08

**Authors:** Sven T. Sowa, Heiner G. Weddeling, Robin Teufel

**Affiliations:** Pharmaceutical Biology, Department of Pharmaceutical Sciences, 27209 University of Basel, Klingelbergstrasse 50, 4056 Basel, Switzerland

## Abstract

Aromatic polyketides from Actinobacteria are a class of natural products with potential for clinical application due to their antibiotic and cytotoxic activities. Among the most complex members are the closely related rubromycins and griseorhodins, which are derived from a pentangular backbone that undergoes extensive oxidative tailoring to afford a characteristic bisbenzannulated spiroketal pharmacophore. Recent insights into the characteristic genetics and enzymology underlying the biosynthesis of these polyketides now enable the bioinformatics-driven search for further congeners. Here, we analyzed 154 available and manually selected rubromycin/griseorhodin biosynthetic gene clusters (BGCs) with the help of the tool BisCEET, which allowed the rapid identification and visualization of “variant-specific tailoring genes” (VSTGs), encoding putative tailoring enzymes required only for individual congeners. This approach revealed a striking BGC variant in the genome of the Actinomycete *Actinacidiphila soli*, predicted to enable the biosynthesis of a glycosylated griseorhodin variant. Following the successful cultivation of the strain, the production of a previously uncharacterized griseorhodin, named ruskamycin, was confirmed that featured a distinctive digitoxose-substituted epoxy-spiroketal pharmacophore and exhibited antibacterial activity against Gram-positive strains.

## Graphical abstract

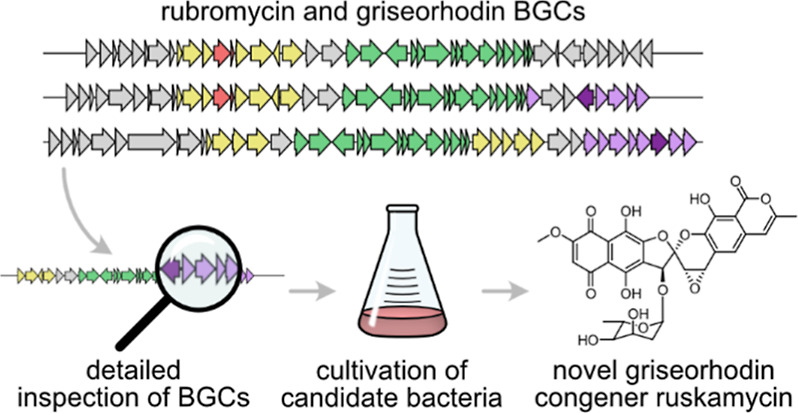

The rubromycins and griseorhodins (distinguished from the rubromycins by a non-oxidized, unsubstituted C11-methyl group, see Figure 1A) are a family of aromatic polyketides produced by Actinobacteria with characteristic spiroketal pharmacophores and a prime example for the diversification of a natural product scaffold resulting in multiple, chemically distinct congeners.^1–9^ During their biosynthesis, the minimal type II polyketide synthase machinery including an acyl-carrier protein (GrhABC for griseorhodin A biosynthesis) and various additional enzymes (e.g., cyclases/aromatases) synthesize the poly-β-ketone (=polyketide) chain and control its subsequent folding and cyclization. Ultimately, these steps afford the characteristic pentangular backbone, which is further modified by the spiroketal-forming tailoring enzymes (i.e., flavoprotein monooxygenases GrhO5 and GrhO6 as well as auxiliary flavoprotein oxidase GrhO1 and acetyltransferase-like GrhJ) and a ketoreductase (GrhO10) to a last shared precursor that is then further converted into the mature rubromycins/griseorhodins (Figure 1A).^6,7,10^ The genes encoding enzymes required for the biosynthesis of the last shared precursor therefore also include tailoring genes (e.g., *grhO1*, *grhO5*, and *grhO6* and their homologues) conserved in all rubromycin/griseorhodin biosynthetic gene clusters (BGCs),^10–13^ which are referred to in this work as core biosynthetic genes (CBGs). However, in the general context of natural product families,^14,15^ tailoring genes may also be restricted to one (or some) of these BGCs (Figure 1) and enable the further structural diversification of late-stage intermediates, e.g., through alkylation, glycosylation, oxygenation, or halogenation.^16,17^ In the case of rubromycins and griseorhodins, such genes are coding for methyltransferases (β-rubromycin), epoxidases (griseorhodin A), or sugar-biosynthetic and -transferring enzymes (heliquinomycin), which are denoted herein as “variant-specific tailoring genes” (VSTGs).

**Figure 1:**
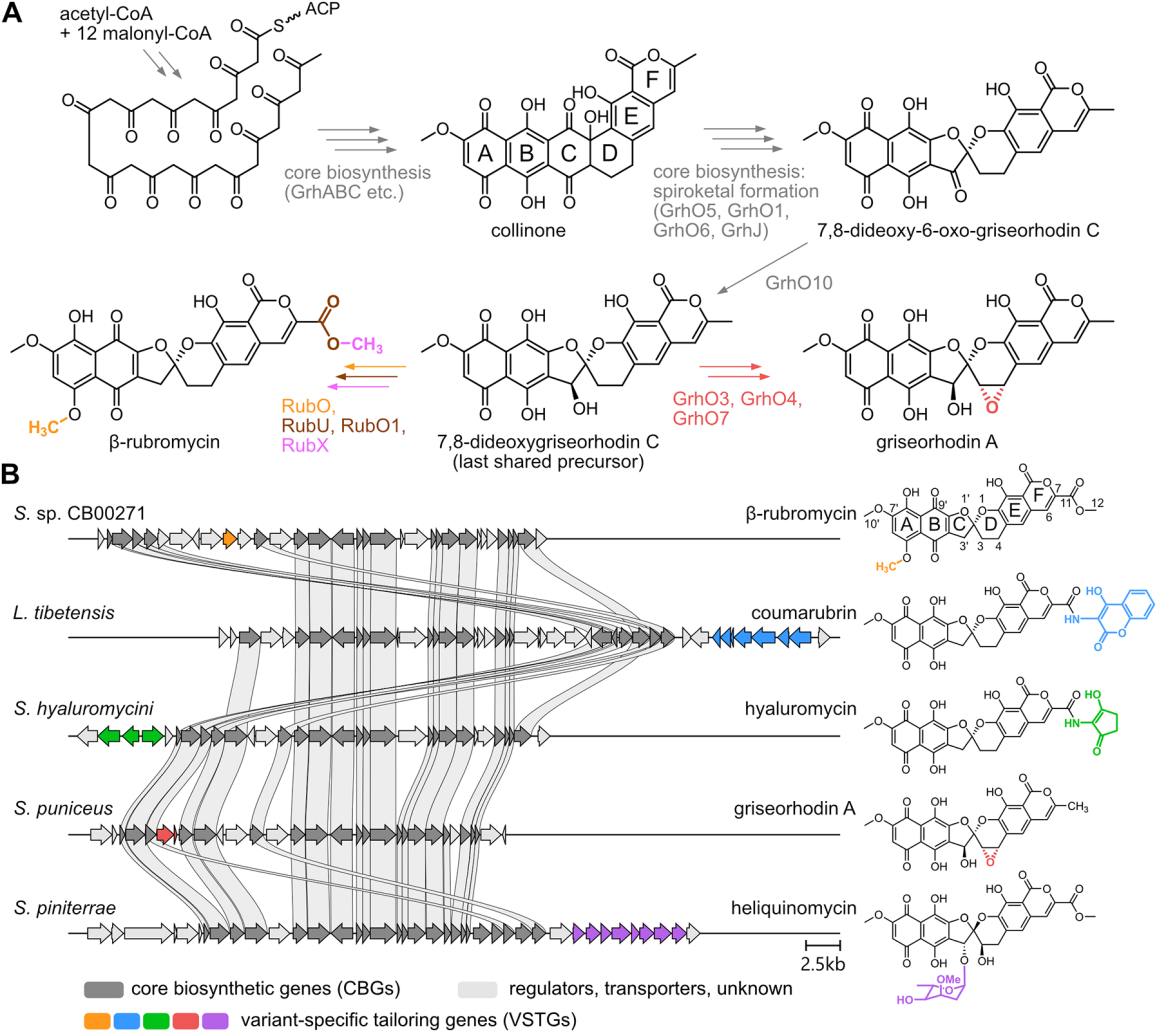
Biosynthesis and BGCs of different rubromycin/griseorhodin family members. (A) Simplified biosynthetic scheme for the formation of rubromycin/griseorhodin polyketides. The presumed last shared precursor is shown, whose biosynthesis relies on CBGs (note that for simplicity, the shown enzymes have the griseorhodin nomenclature “Grh”; homologues in related BGCs may have different annotations, e.g., “Rub” for β-rubromycin). Further modification depends on enzymes encoded by VSTGs found only in the respective BGC variant (see panel B). Final steps leading to griseorhodin A and β-rubromycin are shown as examples. (B) The BGCs from *Streptomyces* sp. CB00271 (β-rubromycin), *Lentzea tibetensis* (coumarubrin), *Streptomyces hyaluromycini* (hyaluromycin), *Streptomyces puniceus* (griseorhodin A), and *Streptomyces piniterrae* (heliquinomycin) are shown. Gene homologues with sequence identities over 40% encoding enzymes involved in the biosynthesis of the rubromycin/griseorhodin core scaffold (CBGs) are indicated by connecting ribbons. Distinct VSTGs and the chemical moieties that are produced or introduced by the corresponding tailoring enzymes share the same color (note that the rubromycin-specific genes encoding late-stage enzymes RubO, RubO1, and RubX are not shown in color).

In order to identify new BGC variants among the rubromycins/griseorhodins, distinct VSTGs associated with CBGs can therefore serve as a key indicator. We thus searched the available bacterial genomes in NCBI GenBank for rubromycin/griseorhodin family BGCs using CluSeek,^18^ which were then analyzed using BisCEET—a bioinformatic tool developed in our group (see the co-published article in this issue: DOI: 10.1021/acs.jnatprod.5c01563). The following search for unique genes possibly involved in the structural diversification of the rubromycin/griseorhodin backbone led to the discovery of the novel family member ruskamycin featuring a digitoxose-substituted griseorhodin scaffold and an epoxy-spiroketal moiety.

## Results and Discussion

In the past, our group has been investigating the biosynthesis of the rubromycin/griseorhodin polyketide family^1,12,13^ and more recently also used a bioinformatics-driven approach to identify the new aminoucoumarin-substituted congener coumarubrin.^1^ To this end, a distinct VSTG encoding an amide-bond synthetase from the hyaluromycin BGC was used (which attaches an aminocyclopentenone moiety to the rubromycin backbone) to find the previously uncharacterized BGC variant of coumarubrin (Figure 1B). In order to allow for a more streamlined and systematic approach, we now developed the bioinformatic tool BisCEET (DOI: 10.1021/acs.jnatprod.5c01563). First, a comprehensive list of rubromycins/griseorhodin BGCs was generated using CluSeek.^18^ To this end, the sequences of CBGs *grhO6* and *grhJ* from griseorhodin A producer *Streptomyces* sp. JP95 were used as input, as the encoded spiroketal-forming enzymes are required only for the biosynthesis of rubromycins/griseorhodins,^6,11–13,19^ but not for other related pentangular polyketides. In total, homologues of both genes (within a distance of 60 kb and >40% sequence identities compared to the input sequences) were found in *N* = 154 genomic sequences, which were collected and then analyzed with BisCEET using the griseorhodin A BGC from *Streptomyces* sp. JP95 as reference.^10^ Then, CBGs (Table S1) as well as known VSTGs (Table S2) in the BGCs of the congeners β-rubromycin, hyaluromycin, griseorhodin A, heliquinomycin, and purpuromycin were marked. The list of rubromycin/griseorhodin-related BGCs was then visualized in BisCEET that enabled the rapid identification and highlighting of putative novel VSTGs. This workflow revealed an interesting candidate BGC variant in *Actinacidiphila soli* (family: Streptomycetaceae; GenBank accession NZ_RJVR01000285.1, coordinates: 5245–51,132), in which the CBGs were directly flanked by putative VSTGs predicted to encode TDP-sugar synthesizing and transferring enzymes (Figure 2A). The only known sugar-substituted rubromycin congener reported to date is heliquinomycin from *Streptomyces piniterrae*, featuring a cymarose moiety.^9^ Comparison of the BGCs from *A. soli* and those of known rubromycin/griseorhodin family members revealed that the cluster is closely related to the BGC of griseorhodin A, most notably containing VSTGs coding for close homologues of GrhO3 and GrhO4 implicated in the installment of the characteristic epoxide moiety of griseorhodin A that is absent in other congeners such as heliquinomycin (Figures 2A, and S1, Table S3). Notably, not all of the sugar-related VSTGs from *S. piniterrae* have homologues in the BGC from *A. soli*; moreover, the encoded glycosyltransferases share only 30% amino acid sequence identity, suggesting different sugar modifications. Closer examination of the *A. soli* sugar-related VSTGs revealed that they share the same composition as reported for the formation and attachment of a 4-*O*-methyl-l-digitoxose moiety in the biosynthesis of polyene macrolide selvamicin from *Pseudonocardia* sp. HH130630-07 (Table S3, Figure S2).^20^ However, the BGC variant from *A. soli* does not encode a sugar methyltransferase (Figure 2B). Based on these results, we predicted that the novel BGC variant most likely enables the production of a griseorhodin congener with an additional (nonmethylated) l-digitoxose moiety attached.

**Figure 2:**
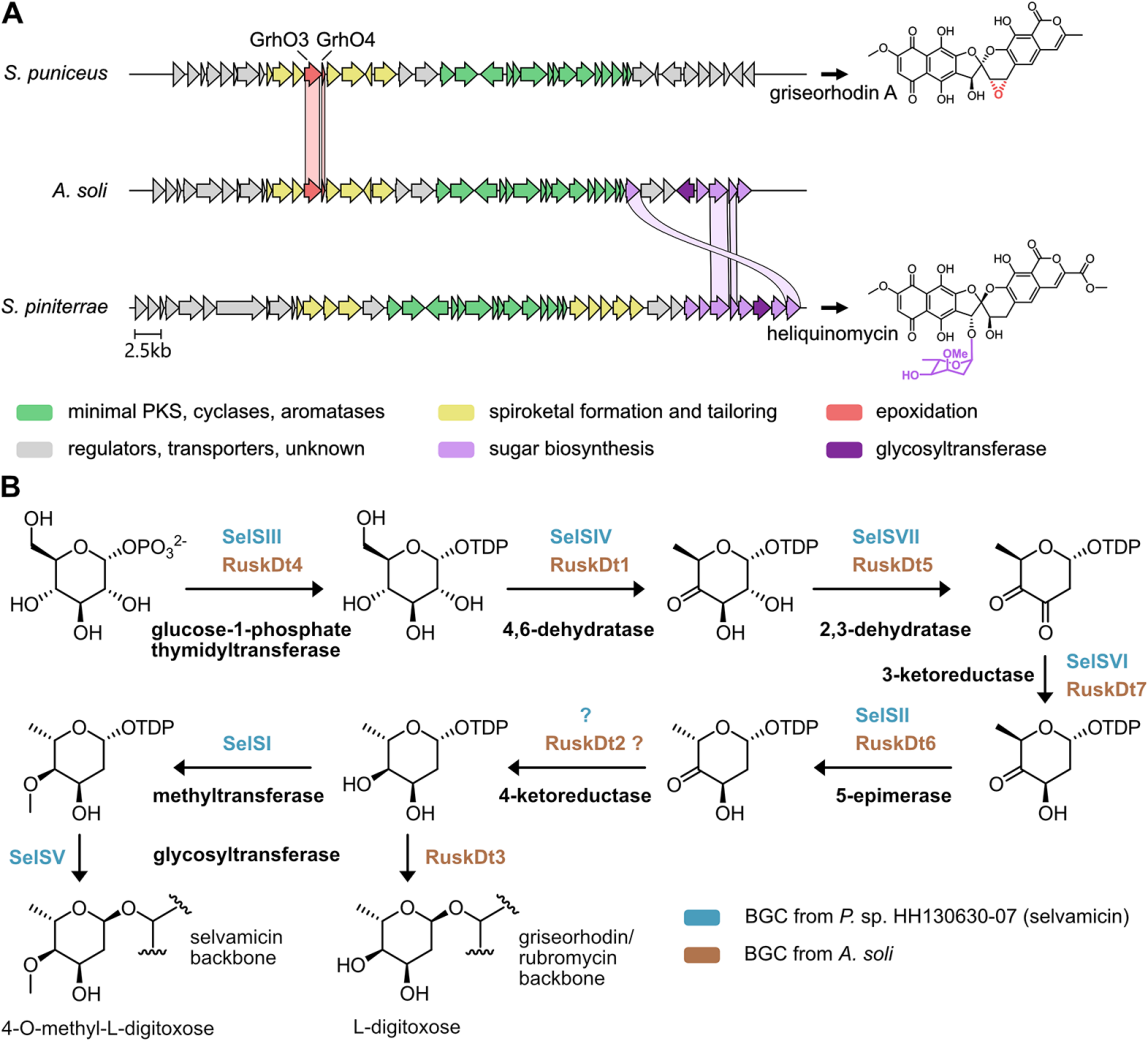
Comparison of the BGCs from *A. soli*with the known rubromycin/griseorhodin producers *S. puniceus*and *S. piniterrae*. (A) The coloration of genes corresponds to functions of the encoded enzymes that were either experimentally validated or predicted based on homology (see legend in figure). Connecting ribbons are shown for homologues of VSTGs involved in epoxidation or sugar biosynthesis that share at least 40% sequence identity. (B) Proposed pathway leading to the biosynthesis and transfer of l-digitoxose in the griseorhodin-related BGC from *A. soli*. For comparison, the pathway leading to the formation of 4-*O*-methyl-l-digitoxose in selvamicin biosynthesis is shown with corresponding enzyme homologues. A gene encoding an enzyme for 4-ketoreduction was not found in the BGC of selvamycin but was suspected elsewhere in the genome. In the BGC variant of *A. soli*, *ruskDt2* is a plausible candidate gene encoding a ketoreductase that may catalyze this step.

Another interesting potential BGC variant was found in *Streptomyces dubilierae*. Here, a rubromycin BGC was located directly adjacent to a gene encoding a nonribosomal peptide synthetase (NRPS)-like protein (Figure S3), which could be involved in the biosynthesis of a NRPS-rubromycin hybrid or supply of a modified starter unit to the PKS.

To experimentally verify the chemical identity of the rubromycins produced by the two BGCs of interest, the strains *A. soli* and *S. dubilierae* were obtained from the Japan Collection of Microorganisms (JCM, RIKEN BioResource Research Center, Japan) and the German Collection of Microorganisms and Cell Cultures (DSMZ, Germany), respectively. The strains were initially cultivated in different media (see the Experimental Section), and cultures were visually inspected for the production of a red pigment. For both strains, the only cultures showing a change in color to purple-red were the ones prepared in GYM media in 100 mL Erlenmeyer flasks with inserted stainless-steel spirals. Ethyl acetate extracts of the cultures were subjected to analysis by LC-DAD and UPLC-HRMS on a reversed-phase C18 column. Peaks with UV/vis-spectra characteristic for rubromycins were detected, showing absorption maxima at 315, 360, and 490 nm. The exact masses determined for the rubromycins produced by *S. dubilierae* corresponded to those of the known β-rubromycin and γ-rubromycin (Figure S4). For the most prominent peak from *A. soli*, *m*/*z* values of 637.12 and 509.07 were measured in negative ion and positive ion mode, respectively (Figure S5). The mass difference of 130 is most likely a result of in-source fragmentation through the cleavage of the O-glycosidic bond in positive ion mode, which is well-described for glycoconjugated compounds^21^ and in line with the expected mass of a griseorhodin containing a single hexose substituent. Based on the HPLC analysis, this compound clearly was the major rubromycin/griseorhodin produced by *A. soli*, suggesting that it represents the final, biologically relevant pathway product rather than a biosynthetic intermediate or degradation product.

The main compounds produced by *S. dubilierae* under the tested culture conditions are apparently known rubromycin congeners, and therefore, no further characterization was pursued (albeit the production of novel, minor constituents cannot be ruled out). Conversely, the compound produced by *A. soli* had a mass different from that of known rubromycin congeners and was thus further investigated. To produce sufficient amounts of this compound for structure elucidation, an upscale in larger culture volumes (200–800 mL) was attempted, resulting, however, in only minor or negligible production (ca. 0.03–0.3 mg/L culture) of the compound of interest (Figure S5). This is likely the result of changes in the oxygen availability in different culture volumes, which is known to be an important factor in the production of bacterial secondary metabolites.^22–24^ Thus, 20 individual 35 mL cultures were grown and combined to a final volume of 750 mL and then centrifuged. The resulting cell pellet was subsequently extracted and purified by preparative HPLC purification, yielding 7 mg of a pure compound. Comprehensive analysis via NMR showed chemical shifts that were in full agreement with the known griseorhodin A backbone containing an additional 2,6-didesoxyhexose moiety linked via an O-glycosidic bond to the hydroxyl group of ring C (Figure 3A, Table S4, Figures S6–S11).

**Figure 3:**
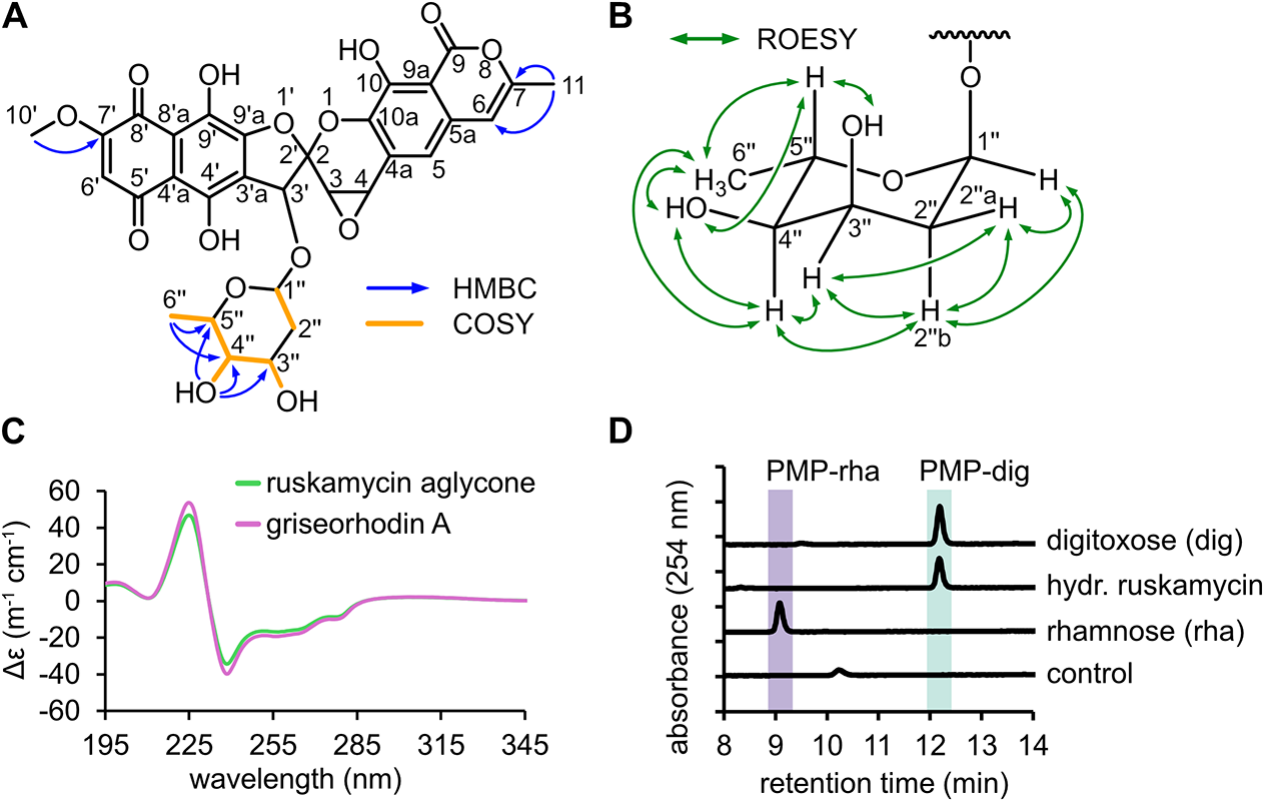
Structure elucidation of ruskamycin. NMR correlations for ruskamycin were determined by (A) HMBC, COSY and (B) ROESY. (C) Circular dichroism spectroscopy was carried out with the ruskamycin aglycone and griseorhodin A. (D) HPLC-analysis of the sugar from hydrolyzed ruskamycin derivatized with PMP. For comparison, PMP-derivatized d-digitoxose (dig) and l-rhamnose (rha) were used. The PMP reaction containing no sugar is shown as control.

Due to the presence of many quaternary carbons in the griseorhodin scaffold, a limited number of COSY and HMBC correlations could be detected. Nonetheless, multiple characteristic signals for the rubromycin backbone can be observed. The methoxy group H_3_-10′, present in all rubromycins, showed an HMBC correlation to C-7′ as seen for hyaluromycin, heliquinomycin, and coumarubrin.^1,5,9^ Diagnostic HMBC correlations as well as ^1^H and DEPTq signals were observed for positions 6, 7, and 11, which were also present in the related dideoxy-griseorhodin C or its hydroxymethyl derivative.^25^ Specifically, protons H_3_-11 showed key HMBC correlations to C-6 and C-7. For the spiroketal position 3′ of ring C, the chemical shifts of ^1^H and ^13^C closely resembled the shifts observed for heliquinomycin.^9^ However, the signals for ^1^H and ^13^C of position 4 of ring D were shifted downfield compared to heliquinomycin due to the epoxide connected to C-3 and C-4, as also observed for griseorhodin A.^9,26^ This is supported by the proton integral of H-4 and the phase of C-4 in the DEPTq. For the sugar moiety, HMBC correlations from H_3_-6″ to C-4″ and C-5″ as well as 4″-OH to C-5″, C-4″ and C-3″ in conjunction with respective COSY correlations confirmed their connectivity. For positions 1″ and 2″, no HMBC correlations were observed; however, ^1^H–^1^H COSY correlations, DEPTq signal of C-2″, and respective ROESY signals of H-1″, H-2″a, and H-2″b strongly suggested their positioning next to C-3″.

The ^1^H spectrum showed three signals of phenolic hydroxyl groups 4′-OH, 9′-OH and 10-OH, as seen for heliquinomycin, griseorhodin A, and other rubromycins.^5,9,25,26^ The ^1^H signals for these three hydroxyl groups could be observed, showing that they are not connected to the glycosyl moiety. The single remaining attachment site is the oxygen located at position 3′, and indeed, a ^1^H signal could not be observed for the corresponding hydroxyl group, as opposed to griseorhodin A^26^ lacking this sugar moiety. Notably, ROESY correlations further indicated that the sugar moiety is digitoxose (Figure 3B). Taken together, the structure shown in Figure 3A is proposed, and the compound was named ruskamycin after “ruska”, the Finnish term for the vibrant change of colors in nature during fall season, reflecting the shift from yellow to orange and red observed in *A. soli* cultures during its production.

To compare the aglycone of ruskamycin with that of griseorhodin A, hydrolysis under mild conditions was conducted to avoid epoxide opening. The resulting ruskamycin aglycone showed an identical retention time to griseorhodin A during HPLC analysis (Figure S12). To compare the absolute configuration, electronic circular dichroism (ECD) spectra of the ruskamycin aglycone and griseorhodin A were recorded (Figure 3C), which showed virtually indistinguishable features, thereby further corroborating identical scaffolds and spiroketal configurations for both griseorhodin congeners. The griseorhodin A ECD spectrum being identical to that of the ruskamycin aglycone was therefore in full agreement with the NMR analysis above. The glycoside moiety of ruskamycin was further compared to a commercial digitoxose standard by derivatization of the sugars with 1-phenyl-3-methyl-5-pyrazolone (PMP), followed by analysis via HPLC-DAD on a C18 column (Figure 3D). A standard of rhamnose, a readily available 6-deoxyhexose structurally related to the 2,6-deoxyhexose digitoxose, was used for comparison. The PMP derivatives of the sugar from ruskamycin and the digitoxose standard showed identical retention times, confirming digitoxose as the sugar moiety of ruskamycin. This was fully consistent with the BGC of ruskamycin from *A. soli* containing homologues of genes encoding l-digitoxose biosynthetic enzymes, previously described in the biosynthesis of selvamicin from *Pseudonocardia* sp. HH130630-07, as pointed out above (Figure 2B).^20^ Interestingly, l-digitoxose from ruskamycin differs structurally from l-cymarose of heliquinomycin only in the absence of a methyl group at the 3″-OH (Figure 2A). However, many homologues responsible for the biosynthesis and transfer of the glycoside moieties in both pathways showed low sequence identities, indicating an example of convergent evolution in which corresponding VSTGs were independently acquired by ancestral rubromycin/griseorhodin producer strains, resulting in near-identical sugar modifications, which could furthermore point toward similar biological roles of the mature products.

Rubromycins/griseorhodins typically display potent antibiotic activity against Gram-positive bacteria. We tested the antimicrobial activity of ruskamycin and determined the minimal inhibitory concentration (MIC) for several bacterial strains and baker’s yeast. Not surprisingly, ruskamycin showed strong activity against the Gram-positive strains *Bacillus subtilis* and *Micrococcus luteus* but no inhibition of yeast or the Gram-negative strains *Escherichia coli* and *Pseudomonas fluorescens* at the tested concentrations (Table 1). Overall, this antimicrobial activity profile appears comparable to other rubromycins.^1^

**Table 1:** Minimal Inhibitory Concentrations of Ruskamycin Measured against Bacterial and Fungal Strains

	Ruskamycin
E. coli	>32 μM
P. fluorescens	>32 μM
M. luteus	1 μM
B. subtilis	0.125 μM
S. cerevisiae	>8 μM

Taken together, a genome mining workflow aided by the visualization software BisCEET was employed to rapidly identify putative VSTGs in BGCs of unexplored bacterial producer strains and, thus, streamline the discovery of previously unknown natural product congeners. As a proof of concept, the workflow was conducted for rubromycin and griseorhodin BGCs, which led to the discovery and characterization of the glycosylated griseorhodin congener ruskamycin in *A. soli*.

## Experimental Section

### General Experimental Procedures

Liquid chromatography–mass spectrometry (LC–MS) measurements were performed on a Shimadzu LCMS-8030 Triple Quad Mass Spectrometer. UV/vis absorption spectra were recorded on a Shimadzu SPD-M20A diode array detector (DAD). Sample analysis was done on a SunFire C18 column (150 × 3 mm ID, 3.5 μm, Waters) equipped with a guard column (10 × 3 mm ID). UV/vis absorption was monitored from 190 to 800 nm during the whole measurement. MS analysis in positive and negative ion modes proceeded with a capillary voltage of 3 kV, 250 °C DL temperature, 400 °C heat block temperature, and 3 L/min nebulizing gas flow. High resolution mass spectrometry was performed on an Agilent UPLC system comprising a 1290 Binary Pump G4220A, Infinity Autosampler G4226A, Infinity Thermostat G1130B, and Thermostated Column Compartment G1316C. The mass detector used was a Q Exactive HF (Thermo Scientific). Chromatographic separation was carried out on an ACQUITY UPLC BEH C18 column (2.1 × 150 mm, 1.7 μm particle size, 130 Å pore size). The mobile phase consisted of water and acetonitrile, each containing 0.1% formic acid. Gradient elution proceeded as follows: initial composition 5% acetonitrile for the first 0.5 min; linear increase to 100% acetonitrile for 15 min; a wash step at 100% acetonitrile maintained for 2.3 min; followed by re-equilibration to 5% acetonitrile over 3 min before the next injection.

### Bioinformatic Tools

CluSeek^18^ was used to collect BGCs from genomic sequences deposited in NCBI GenBank. Files with BGCs were exported in the GenBank file format. BisCEET (10.1021/acs.jnatprod.5c01563) was used to compare rubromycin/griseorhodin BGCs and search for potential novel VSTGs in proximity to rubromycin CBGs. Figures comparing BGCs were generated with clinker as part of the CAGECAT package.^27^

### Cultivation of *S. dubilierae*

*S. dubilierae* (DSM 41921) was obtained from the German Collection of Microorganisms and Cell Cultures (DSMZ, Germany). The freeze-dried culture was rehydrated in GYM media (4 g/L glucose, 4 g/L yeast extract, 10 g/L malt extract, pH 7.2) for 30 min. The rehydrated cell material was transferred into multiple liquid media and onto solid media for cultivation tests. Liquid cultures were prepared in 100 mL Erlenmeyer flasks with a stainless-steel metal spring containing 10 mL of GYM, MYM (4 g/L maltose, 4 g/L yeast extract, 10 g/L malt extract, pH 7.2), or TSB medium (CASO-Bouillon (Ph. Eur./USP, ISO 10273)). The flasks were incubated at 115 rpm and 28 °C in a (Infors HT Multitron Triple Incubator Shaker). For cultivation on Agar plates, MYM or GYM plates (respective media with 17.5 g/L of agar) or SFM agar (20 g/L soy flour, 20 g/L mannitol, 20 g/L agar) were inoculated and incubated at 28 °C. Red pigment formation was observed in GYM medium within 3 days and increased up until 6 days of cultivation. For analysis the whole culture was extracted consecutively with n-butanol, ethyl acetate, and ethyl acetate containing 1% (v/v) formic acid. The last acidified fraction contained most of the red pigment, which turned out to be mostly β-rubromycin (see the Supporting Information).

### Cultivation of *A. soli* and Production of Ruskamycin

*A. soli* LAM7114 (JCM 32822) was obtained from the Japan Collection of Microorganisms (JCM, RIKEN BioResource Research Center, Japan). The freeze-dried culture was rehydrated in ISP-1 medium containing MgSO_4_ (5 g/L tryptone, 3 g/L Yeast extract, 2 g/L MgSO_4_·7H_2_O, pH 7.2) for 30 min at ambient temperature and streaked out and maintained on yeast extract-starch agar (2 g/L yeast extract, 10 g/L soluble starch, 15 g/L agar) at 28 °C. For production of glycerol stocks, a 100 mL Erlenmeyer flask containing 35 mL of GYM media (4 g/L glucose, 4 g/L yeast extract, 10 g/L malt extract, pH 7.2) and a stainless-steel spring was inoculated with a colony of *A. soli* grown on YS agar. Liquid cultures were grown in an incubator shaker (Infors HT Multitron Triple Incubator Shaker) at 30 °C and 160 rpm for 3 days. Glycerol stocks were prepared by mixing cells with 50% (v/v) glycerol to a final concentration of 30% (v/v) glycerol. Initial cultivation tests were done in GYM, MYM, TSB, and 2xYT (16 g/L tryptone, 10 g/L yeast extract, 5 g/L NaCl) media. For the production of ruskamycin, liquid cultures were prepared as described above, inoculated with 20 μL of glycerol stock, and incubated for 7–14 days. Cultures were harvested by centrifugation at 8000*g* for 1 h. The supernatant was discarded, and the cell pellets were frozen at −20 °C.

### Cultivation of *S. puniceus* and Production of Griseorhodin A

Precultures of *S. puniceus* DSM 41106 were inoculated from spore stocks into 50 mL of GYM medium within a 250 mL baffled flask and shaken for 24 h at 28 °C and 130 rpm in an Infors HT Multitron Triple Incubator Shaker. Main cultures of 200 mL of GYM in 1 L baffled flasks were inoculated with 8 mL of the preculture and incubated under the same conditions for 5 days. Cultures were harvested by centrifugation at 8000*g* for 20 min and supernatant and cell mass handled separately for extraction and purification of griseorhodin A.

### Isolation of Ruskamycin

Frozen cell pellets from ca. 750 mL of culture were lyophilized. The resulting 3 g of dry mycelia was finely ground with a pestle and mortar and rehydrated in 160 mL of water. 800 mL of ethyl acetate was added to the slurry and it was stirred for 2 h. The mixture was allowed to settle, and the bright red organic phase was decanted and filtered, after which the ethyl acetate was removed under reduced pressure. The remaining slurry was extracted another four times this way. The crude extract was dissolved in DMSO to a concentration of 80 mg/mL and purified by preparative HPLC on an Agilent 1100 HPLC system with a SunFire C18 column (250 × 10.0 mm). As the solvent system, a mixture of water containing 1% (v/v) formic acid (solvent A) and acetonitrile containing 1% (v/v) formic acid (solvent B) was used. The run was performed with an initial 2 min of 45% B followed by a gradient of 45% B to 60% B over the course of 13 min. Each run was followed by a 100% B wash phase and re-equilibration. Collected fractions containing ruskamycin were combined, and acetonitrile was removed under reduced pressure. The remaining water and formic acid were removed by lyophilization.

### Isolation of Griseorhodin A

The culture supernatant was acidified to pH between 1 and 2 with HCl. Cells were resuspended in a minimum amount of water and disrupted via sonication Fisherbrand model 505 Sonic Dismembrator equipped with a 13 mm probe using a total on time of 15 min with 15 s on and 3 s off at 40% amplitude, before acidification with 5% HCl to a pH of 1–2. The supernatant and cells were extracted separately with an equal volume of ethyl acetate three times. Combined organic extracts were evaporated under reduced pressure to obtain the raw extract. The combined raw extract was washed in a 250 mL round-bottom flask by addition of 5 mL of hexane, sonication, and following settling of insoluble particles before removing of the supernatant. Following, the washing was repeated with 3 mL of methanol.

The washed raw extract was further purified by preparative HPLC on an HPLC system (Agilent Technologies, Santa Clara, CA, USA) containing a binary pump (1260 Prep Bin Pump) and a PDA detector (1100 Series). For separation a SunFire Prep C18 OBD column (5 μm, 150 × 30 mm i.d., Waters, Milford, MA, USA) and a C18 Prep Guard Cartridge (10 × 30 mm i.d.) were used. The sample was dissolved in a 1:1 (v/v) mixture of DMSO and methanol to a concentration of 20 mg/mL, sonicated, and centrifuged at 3432*g* before injection by the 1290 Infinity II Valve Drive manual injection system (Agilent Technologies). As a mobile phase, water (solvent A) and acetonitrile (solvent B) each containing 0.1% formic acid as a modifier, were used. The method for separation at 20 mL/min flow rate was 50% solvent B for the initial 7 min with subsequent gradient increase to 70% B at 14 min with a following wash at 100% B for 5 min before reequilibration for the next run.

### Hydrolysis of Ruskamycin

Ruskamycin stock solution (50 mM in DMSO) was diluted 1:200 in 6 mL of 0.1 M H_2_SO_4_ solution to a concentration of 250 μM. The mixture was divided into 500 μL aliquots in 2 mL Eppendorf tubes and incubated at 50 °C for 3.5 h in a thermoshaker (BioShake iQ). Extraction of the aglycones and remaining unreacted compounds was done two times with 6 mL of ethyl acetate. The ethyl acetate phase was dried under N_2_-flow and the ruskamycin aglycone was purified by preparative HPLC as described above for the isolation of ruskamycin. The aqueous phases containing the hydrolyzed sugars were neutralized with Ba­(OH)_2_. The resulting BaSO_4_ precipitate was pelleted by centrifugation at 3000*g* for 10 min, and the clear supernatants were lyophilized. The residues obtained were dissolved in 8 mL of MeOH, filtered, and dried under N_2_-flow.

### Carbohydrate Derivatization with 1-Phenyl-3-methyl-5-pyrazolone

Carbohydrates from the neutralized aqueous phases from the hydrolysis of ruskamycin were derivatized with 1-phenyl-3-methyl-5-pyrazolone (PMP). For derivatization, 100 μL of aqueous ruskamycin hydrolysate or commercial carbohydrate standards (250 μg/mL d-digitoxose or l-rhamnose), 100 μL of 0.3 M NaOH, and 100 μL of 0.5 M PMP in MeOH were mixed and incubated in a thermoshaker (BioShake iQ) for 90 min at 70 °C and 1200 rpm. The reactions were neutralized by addition of 100 μL of 0.3 M HCl. Unreacted PMP was extracted four times with 1 mL of chloroform. The aqueous supernatant was analyzed by HPLC-DAD and UPLC-HRMS.

### LC–MS and HPLC-DAD Measurements

Elution of rubromycins proceeded with 45% acetonitrile in water with 0.1% (v/v) formic acid for the first 5 min, followed by a 20 min gradient up to 100% acetonitrile with 0.1% (v/v) formic acid. Elution of PMP-sugar derivatives proceeded with 20% acetonitrile in water with 0.1% (v/v) formic acid for the first 2 min, followed by a 15 min gradient up to 35% acetonitrile with 0.1% (v/v) formic acid. After that, a column wash at 100% acetonitrile and subsequent equilibration to the starting condition for 5 min followed.

### NMR Measurements

Purified ruskamycin was dissolved in 375 μL of deuterated DMSO and transferred into a 5 mm NMR tube. The spectra were recorded on a Bruker Avance III NMR spectrometer operating at 500.13 and 125.77 MHz for ^1^H and ^13^C nuclei, respectively. ^1^H, ^13^C, HSQC, HMBC, ROESY, and COSY spectra were recorded at 23 °C on a BBO probe.

### Electronic Circular Dichroism Measurements

Electronic circular dichroism (ECD) spectra were recorded on a Chirascan Plus CD spectrometer (Applied Photophysics Ltd., Surrey, UK) equipped with a thermostated cuvette holder. Measurements were performed at 25 °C in a wavelength range from 195 to 400 nm (1 nm bandwidth, 2 s per data point). Samples were dissolved in HPLC-grade acetonitrile at a concentration of 0.083 mg/mL and placed in 1 mm path-length quartz cuvettes (Hellma GmbH, Müllheim, Germany). Data were recorded and analyzed with the Pro-Data V2.4 software (Applied Photophysics).

### Antimicrobial Activity

For testing of minimal inhibitory concentrations (MICs), the following strains were obtained from DSMZ: *B. subtilis* (DSM 23778), *M. luteus* (DSM 20030), *E. coli* (DSM 498), *P. fluorescens* (DSM 50090), and *S. cerevisiae* (DSM 1334). MICs of ruskamycin were determined according to the Clinical and Laboratory Standards Institute (CLSI) guidelines with some alterations as previously described.^1^ The incubation temperature for S. cerevisiae was 30°C.

## Supplementary Material





## Data Availability

The data not included within the article or the Supporting Information will be shared on reasonable request to the corresponding author. Raw NMR data are available as a supplemental file.

## Notes

GenBank accession identifiers related to genome mining efforts, description of the ruskamycin BGC,NMR spectra of ruskamycin, and comparative HPLC chromatograms (PDF)
Raw NMR data of ruskamycin (ZIP)
