# Supplementary material for: Genome Mining-Guided Discovery of the Glycosylated Griseorhodin Congener Ruskamycin
Source: J Nat Prod. 2026 May 8;89(5):1399–406. doi: 10.1021/acs.jnatprod.6c00190 (PMC13200261; doi:10.1021/acs.jnatprod.6c00190)
Supplement: Supplementary file 1 [file np6c00190_si_001.pdf]

Supporting information

Genome Mining-Guided Discovery of the Glycosylated Griseorhodin Congener Ruskamycin

Sven T. Sowa[1], Heiner G. Weddeling[1] and Robin Teufel[1*]

[1] Pharmaceutical Biology, Department of Pharmaceutical Sciences, University of Basel, Klingelbergstrasse 50, 4056 Basel (Switzerland).

*E-mail: robin.teufel@unibas.ch

Contents

Table S1: Rubromycin/griseorhodin core biosynthetic genes from *Streptomyces* sp. JP95 used as input in BisCEET....3

Table S2: Additional biosynthetic genes from rubromycin/griseorhodin congeners used as input in BisCEET....4

Figure S1: Detailed view of the BGC of ruskamycin from *Actinacidiphila soli*....5

Figure S2: Comparison of the genes related to L-digitoxose biosynthesis and transfer from the BGCs of selvamycin and ruskamycin....8

Figure S3: Biosynthetic gene cluster of *Streptomyces dubilierae*....9

Figure S4: UPLC-HRMS and HPLC-DAD analysis of the raw extract from *Streptomyces dubilierae*....10

Figure S5: Analysis of raw extracts from cultures of *Actinacidiphila soli*. 11

Table S4: NMR shifts for ruskamycin....12

Figure S6: ^{1}H NMR spectrum of ruskamycin. The spectrum was recorded in DMSO-d_6, at 500 MHz. 13

Figure S7: ^{13}C-DEPTq NMR spectrum of ruskamycin. The spectrum was recorded in DMSO-d_6 at 125 MHz....14

Figure S8: HSQC-DEPT spectrum of ruskamycin (DMSO-d_6, 500 MHz)....15

Figure S9: HMBC spectrum of ruskamycin (DMSO-d_6, 500 MHz)....16

Figure S10: ROESY spectrum of ruskamycin (DMSO-d_6, 500 MHz)....17

Figure S11: COSY spectrum of ruskamycin (DMSO-d6, 500 MHz)....18

Figure S12: HPLC-DAD analysis of ruskamycin and its aglycone in comparison with griseorhodin A. 19

Sequences used as markers in CluSeek for retrieval of rubromycin/griseorhodin gene clusters:

GrhO6 (*Streptomyces* sp. JP95, GenBank identifier: AAM33673.1):

MPDTKGTTDTIDTPGFDFDVIIVGGGPVGMLLASELRIGRAKAVVLEKLTERTPHSKAFGLHA
RSLESLDRRGLADRFREGARSWNNGHFAGLDVWVDFSLLDSAHNYALLSEQTRTERLLEE
RAEEFGCTIRRGHEVTAVRQDEDGAEVDVTGPDGPYTLRARYVVGTDGGRSLVRRSAGIA
FPGTGGRVTARLADVVLADRENAPMGMERTERGLLFCVPLDDTYHRVATFDYEQGKEAGS
ELGFEEFKDSVRAIWGDDMGASEPRWLSWFTDSACQAETYRAGRILLAGDAAHTHFPVGG
QGVNLGLQDALNLGWKLCADINGWAGEGLLDTYDAERQEPARQVLANTRAQIALMNPDPY
VTQLRELFQDLMRKDQVNHHIAEMLSGVRVRYDLPGPAHRLLGDFARDLRLETEEGPRTLP
KFLRRGNFVLLDLAGRPEIAEEYAKWAAPLDWAGRVRHLVATCEDEPELAGALIRPDGYVA
WAADRDASPAEIAEGLRTAIETWAGITDPTRAVFS

GrhJ (*Streptomyces* sp. JP95, GenBank identifier: AAM33662.1):

MSLELPGDPPVSPALLARLLQRRHGADRIVLAAFDGDRPAGCTKLGLDLGVPDGPGHGSL
WVFPGFRRRGAGTALVSAARTELRARGRDLLMVDAPHTPAAERFAAACGAEALGTNLRNR
LLLKGPGRAGLDAAAGRAVPGHRLVHWSGRCPDELVDSYARAWGALETAANGQAAVRRPA
AADVRAREAEAERAGHRPYVTAAVDTAGEVVGYATLFVRDSPMADAGETLVVAGRRRRGL
GSWTKAALLSRAAAENPHLALVQAWNDVRDEASLALNRRLGFVGDSSWTTNAVKA

Table S1: Rubromycin/griseorhodin core biosynthetic genes from *Streptomyces* sp. JP95 used as input in BisCEET.

Core biosynthetic genes
**GrhI** (AAM33661.1)
**GrhO1** (AAM33667.1)
**GrhJ** (AAM33662.1)
**GrhO5** (AAM33672.1)
**GrhR3** (AAM33681.1)
**GrhO6** (AAM33673.1)
**GrhO7** (AAM33674.1)
**GrhL** (AAM33664.1)
**GrhO8** (AAM33675.1)
**GrhO9** (AAM33676.1)
**GrhM** (AAM33665.1)
**GrhN** (AAM33666.1)
**GrhP** (AAM33677.1)
**GrhQ** (AAM33678.1)
**GrhS** (AAM33682.1)
**GrhA** (AAM33653.1)
**GrhB** (AAM33654.1)
**GrhO10** (AAM33668.1)
**GrhU** (AAM33683.1)
**GrhV** (AAM33684.1)

Table S2: Additional biosynthetic genes from rubromycin/griseorhodin congeners used as input in BisCEET.

**Griseorhodin A (*Streptomyces* sp. JP95) - Epoxidation**
AAM33670.1
AAM33671.1
**Hyaluromycin (*Streptomyces hyaluromycini*) – C_5N biosynthesis and transfer**
WP_167449844.1
WP_089099035.1
WP_089099034.1
**β-Rubromycin (*Streptomyces* sp. CB00271) – Methylation and methyl ester introduction**
QNQ38831.1
QNQ35677.1
QNQ35678.1
**Heliquinomycin (*Streptomyces piniterrae*) – Cymarose biosynthesis and transfer**
TJZ52148.1
TJZ52149.1
TJZ52150.1
TJZ52151.1
TJZ52222.1
TJZ52152.1
TJZ52153.1
TJZ52154.1
TJZ52155.1
**Purpuromycin (*Actinoplanes ianthinogenes*) – Hydroxylation**
GGR05530.1

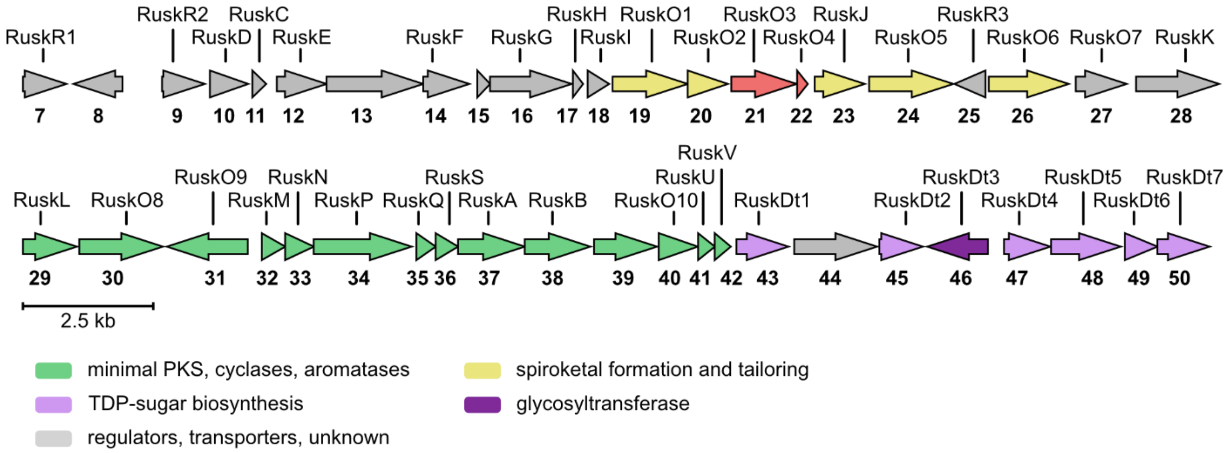

Figure S1: Detailed view of the BGC of ruskamycin from *Actinacidiphila soli*. Genes in the cluster are named corresponding to the nomenclature of homologs found in the biosynthetic gene cluster of griseorhodin A from *Streptomyces* sp. JP95. Putative digitoxose biosynthesis related genes were labelled "RuskDt". Numbering (7-50) was done according to the position of the genes in the corresponding contig (*Actinacidiphila soli* strain LAM7114 Z_ctg007, NZ_RJVR01000285).

Table S3: List of genes in the biosynthetic gene cluster of ruskamycin from *A. soli*. Comparison with homologs of genes from the BGCs of *S.* sp. JP95 (griseorhodin A, "Grh") or from *Pseudonocardia* sp. HH130630-07 (selvamicin, "Sel") were done. Numbering of the genes from the ruskamycin BGC is identical to Figure S2. Sequence identities between homologs were determined by global pairwise sequence alignment. The putative function was assigned based on the function reported in the respective Grh or Sel BCGs. Putative functions of genes with no homologs in Grh or Sel gene clusters were assigned based on the annotation of these genes.

Ruskamycin BGC genes (proposed name)	Homolog from Grh or Sel BGC (% sequence identity)	Putative function
7 (RuskR1)<br>WP_127359069.1	GrhR1 (54%)<br>AAM33679.1	Transcription regulator
8<br>WP_127359013.1	-	ScbA/BarX family gamma-butyrolactone biosynthesis protein
9 (RuskR2)<br>WP_127359014.1	GrhR2 (73%)<br>AAM33680.1	Transcription regulator
10 (RuskD)<br>WP_127359015.1	GrhD (53%)<br>AAM33656.1	Thioesterase II family protein
11 (RuskC)<br>WP_127359016.1	GrhC (59%)<br>AAM33655.1	Phosphopantetheine-binding protein
12 (RuskE)<br>WP_127359017.1	GrhE (44%)<br>AAM33657.1	Aromatase/cyclase
13<br>WP_127359018.1	-	Acyl-CoA dehydrogenase
14 (RuskF)<br>WP_127359019.1	GrhF (51%)<br>AAM33658.1	4'-phosphopantetheinyl transferase family protein
15<br>WP_240677587.1	-	DUF6059 family protein
16 (RuskG)<br>WP_127359020.1	GrhG (84%)<br>AAM33659.1	Acyl-CoA carboxylase subunit beta
17 (RuskH)<br>WP_127359021.1	GrhH (31%)<br>AAM33660.1	Acyl-CoA carboxylase subunit epsilon
18 (RuskI)<br>WP_127359022.1	GrhI (81%)<br>AAM33661.1	Hypothetical protein
19 (RuskO1)<br>WP_127359023.1	GrhO1 (72%)<br>AAM33667.1	Spiroketal formation
20 (RuskO2)<br>WP_127359024.1	GrhO2 (81%)<br>AAM33669.1	Spiroketal formation
21 (RuskO3)<br>WP_127359025.1	GrhO3 (85%)<br>AAM33670.1	Epoxidation
22 (RuskO4)<br>WP_127359026.1	GrhO4 (63%)<br>AAM33671.1	Epoxidation
23 (RuskJ)<br>WP_127359027.1	GrhJ (61%)<br>AAM33662.1	Spiroketal formation
24 (RuskO5)<br>WP_127359028.1	GrhO5 (73%)<br>AAM33672.1	Spiroketal formation
25 (RuskR3)<br>WP_127359029.1	GrhR3 (76%)<br>AAM33681.1	Transcription regulator
26 (RuskO6)<br>WP_127359030.1	GrhO6 (74%)<br>AAM33673.1	Spiroketal formation
27 (RuskO7)<br>WP_127359031.1	GrhO7 (82%)<br>AAM33674.1	Epoxidation
28 (RuskK)<br>WP_240677588.1	GrhK (66%)<br>AAM33663.1	MDR family MFS transporter

Table S3: Continued.

29 (RuskL)	GrhL (81%)	Rubromycin core biosynthesis

WP_127359032.1	AAM33664.1	
30 (RuskO8)<br>WP_127359033.1	GrhO8 (76%)<br>AAM33675.1	Rubromycin core biosynthesis
31 (RuskO9)<br>WP_127359034.1	GrhO9 (80%)<br>AAM33676.1	Rubromycin core biosynthesis
32 (RuskM)<br>WP_127359035.1	GrhM (86%)<br>AAM33665.1	Rubromycin core biosynthesis
33 (RuskN)<br>WP_127359036.1	GrhN (50%)<br>AAM33666.1	Rubromycin core biosynthesis
34 (RuskP)<br>WP_127359037.1	GrhP (83%)<br>AAM33677.1	Rubromycin core biosynthesis
35 (RuskQ)<br>WP_127359038.1	GrhQ (83%)<br>AAM33678.1	Rubromycin core biosynthesis
36 (RuskS)<br>WP_127359039.1	GrhS (74%)<br>AAM33682.1	Rubromycin core biosynthesis
37 (RuskA)<br>WP_127359040.1	GrhA (84%)<br>AAM33653.1	Rubromycin core biosynthesis
38 (RuskB)<br>WP_127359041.1	GrhB (77%)<br>AAM33654.1	Rubromycin core biosynthesis
39 (RuskT)<br>Id missing in GenBank	GrhT (84%)<br>AAM33685.1	Rubromycin core biosynthesis
40 (RuskO10)<br>WP_127359043.1	GrhO10 (85%)<br>AAM33668.1	Rubromycin core biosynthesis
41 (RuskU)<br>WP_127359044.1	GrhU (77%)<br>AAM33683.1	Rubromycin core biosynthesis
42 (RuskV)<br>WP_127359045.1	GrhV (80%)<br>AAM33684.1	Rubromycin core biosynthesis
43 (RuskDt1)<br>WP_127359046.1	SelSIV (53%)<br>ANY10595.1	TDP-L-digitoxose biosynthesis
44<br>WP_206305654.1	-	MDR family MFS transporter
45 (RuskDt2)<br>WP_127359048.1	-	TDP-L-digitoxose biosynthesis<br>Ketoreductase
46 (RuskDt3)<br>WP_127359049.1	SelSV (30%)<br>ANY10594.1	L-digitoxose transfer<br>Glycosyltransferase
47 (RuskDt4)<br>WP_127359050.1	SelSIII (60%)<br>ANY10596.1	TDP-L-digitoxose biosynthesis
48 (RuskDt5)<br>WP_127359051.1	SelVII (40%)<br>ANY10592.1	TDP-L-digitoxose biosynthesis
49 (RuskDt6)<br>WP_127359052.1	SelSII (66%)<br>ANY10597.1	TDP-L-digitoxose biosynthesis
50 (RuskDt7)<br>WP_240677589.1	SelSVI (40%)<br>ANY10593.1	TDP-L-digitoxose biosynthesis

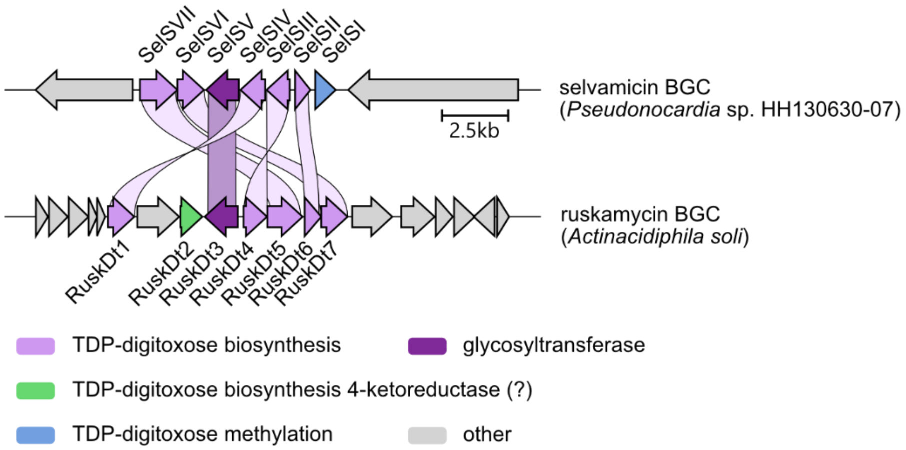

Figure S2: Comparison of the genes related to L-digitoxose biosynthesis and transfer from the BGCs of selvamycin and ruskamycin. Only the section relevant for sugar biosynthesis and transfer in each BGC is shown. Connecting ribbons indicate homologs that share 40% or higher sequence identity (see Table S3), with exception of the glycosyltransferase (30% sequence identity).

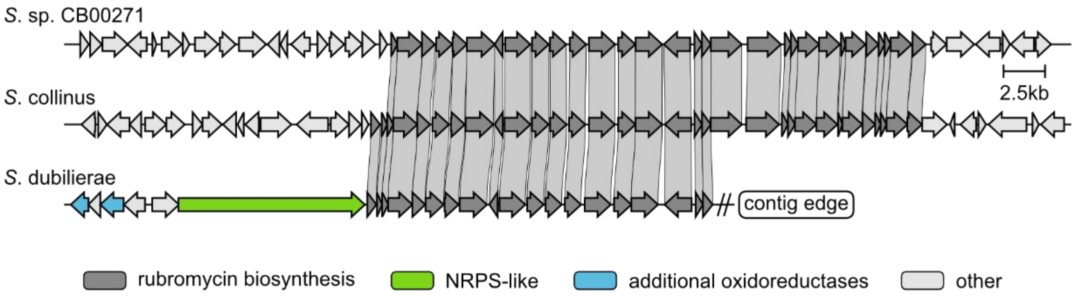

Figure S3: Biosynthetic gene cluster of *Streptomyces dubilierae*. The gene cluster of *S. dubilierae* is on the edge of the contig. It is closely related with BGCs from *Streptomyces* sp. CB00271 and *Streptomyces collinus*, which were reported to produce β-rubromycin and γ-rubromycin. Connecting ribbons are shown between homologs. The BGC from *S. dubilierae* is flanked by genes encoding an NRPS-like protein and additional oxidoreductases, which could potentially serve as VSTGs in the biosynthesis of a novel rubromycin congener.

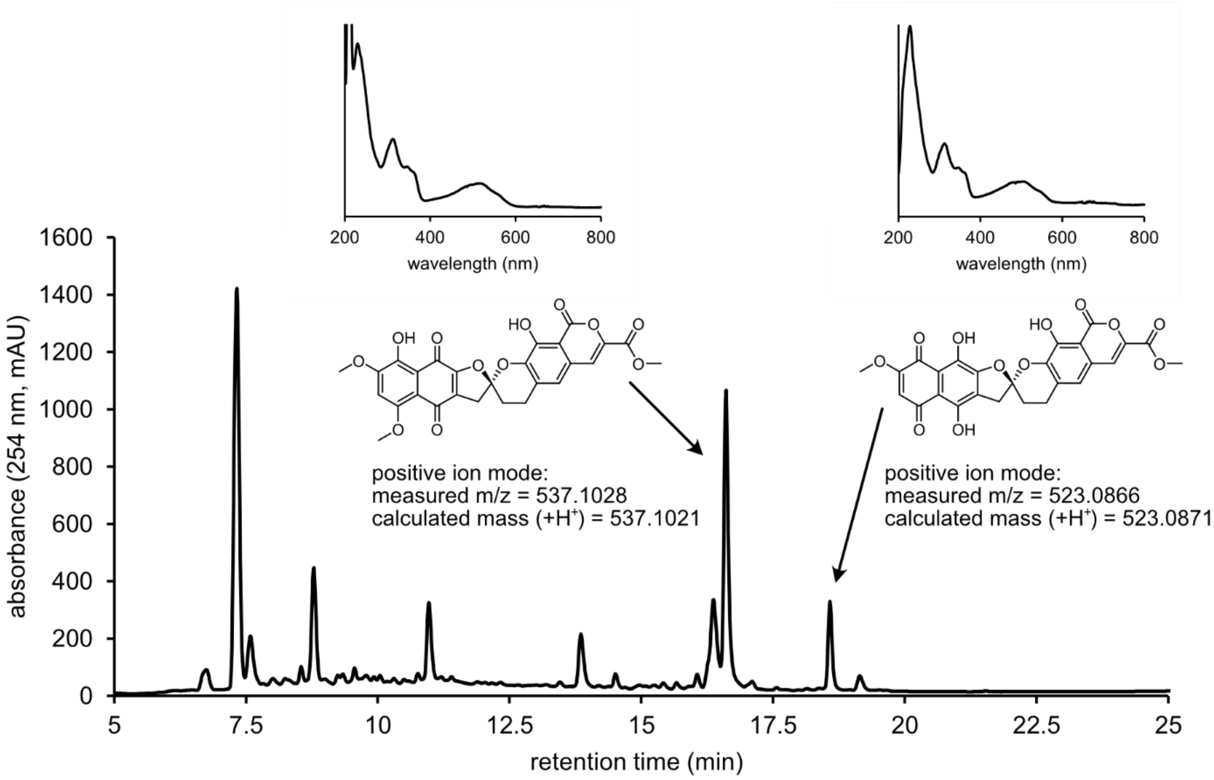

Figure S4: UPLC-HRMS and HPLC-DAD analysis of the raw extract from *Streptomyces dubilierae*. UV/Vis absorption spectra are shown for the peaks with exact masses corresponding to the known β-rubromycin and γ-rubromycin.

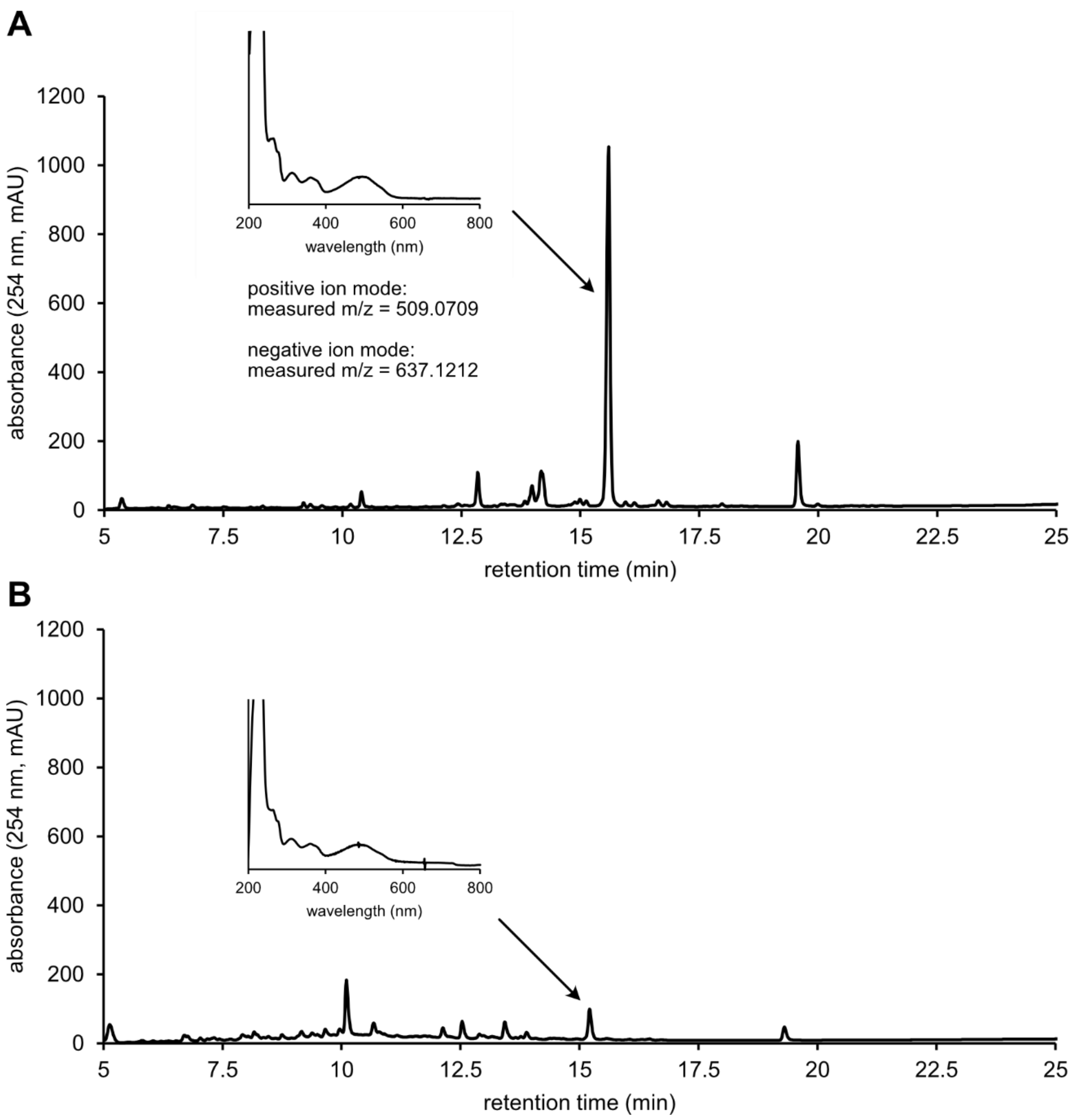

Figure S5: Analysis of raw extracts from cultures of *Actinacidiphila soli*. (**A**) UPLC-HRMS and HPLC-DAD analysis of the ethyl acetate extract of *A. soli* sampled (1 mL) from a 35 mL culture. The UV/Vis absorption spectrum for the highest peak is shown. Exact masses were measured in positive and negative ion modes. (**B**) HPLC-DAD analysis of the ethyl acetate extract of *A. soli* sampled (1 mL) from a 400 mL culture. The amount injected was 3 times higher compared to the extract from the 35 mL culture. The peak corresponding to the rubromycin is highlighted with an arrow and the corresponding UV/Vis absorption spectrum.

Table S4: NMR shifts for ruskamycin. NMR ^{1}H and ^{13}C chemical shifts.

Nr.	δ_C	δ_H
3	49.21	4.48
4	54.80	4.35
5	115.82	7.13
5a	129.58	
6	103.78	6.48
7	153.34	
9	165.16	
10-OH		10.78
10a	135.14	
11	18.75	2.22
3’	78.85	5.49
4’-OH		11.70
4’a	106.69	
6’	109.86	6.38
7’	160.61	
8’a	120.63	
9’-OH		13.40
9’a	148.69	
10’	57.10	3.911
1”	96.66	
2”	35.27	2.02
		1.95
3”	65.61	3.79
3”-OH		3.94
4”	72.25	3.12
4”-OH		4.62
5”	64.02	4.33
6”	17.78	1.24

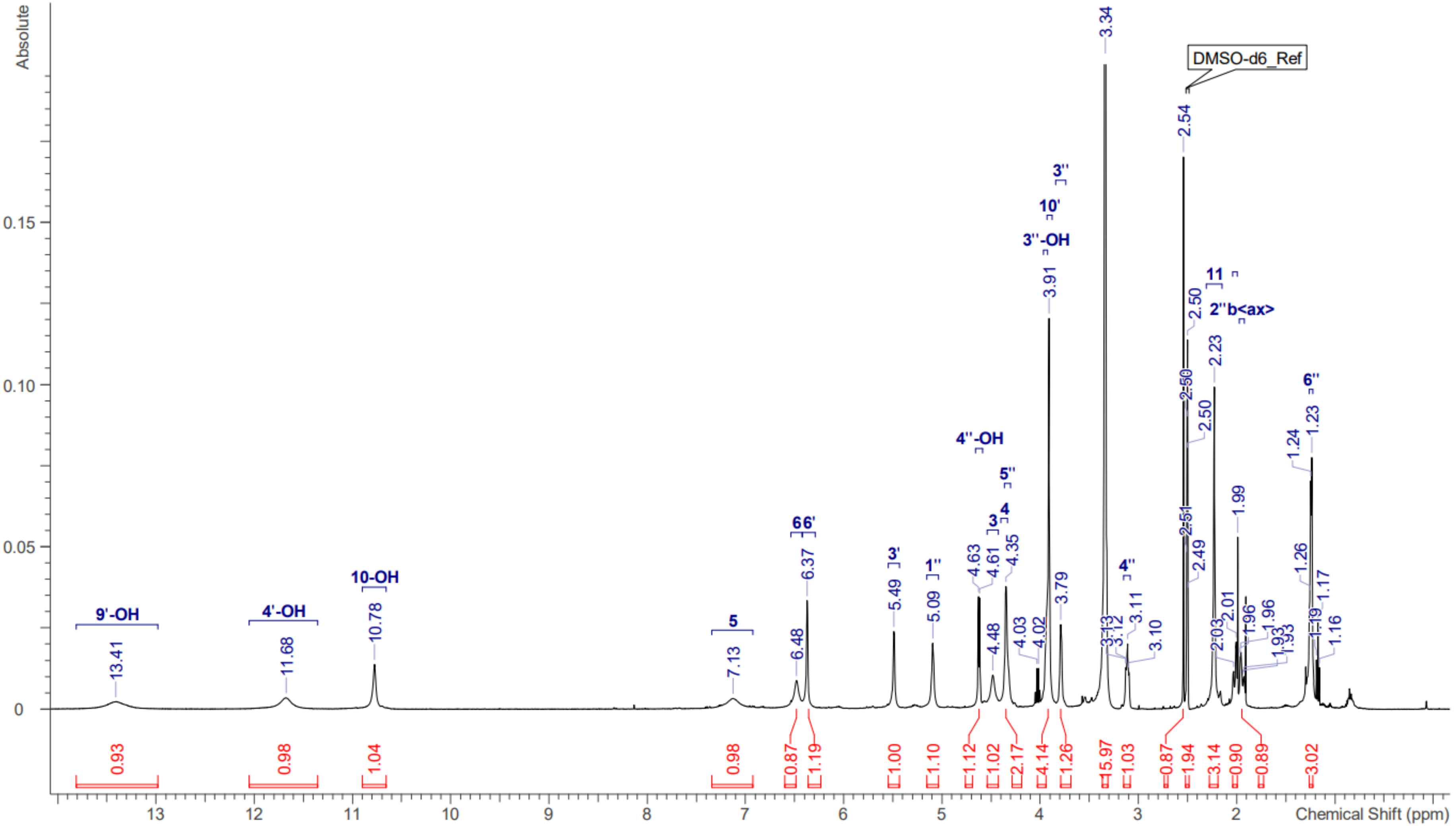

Figure S6: ^{1}H NMR spectrum of ruskamycin. The spectrum was recorded in DMSO-d6, at 500 MHz.

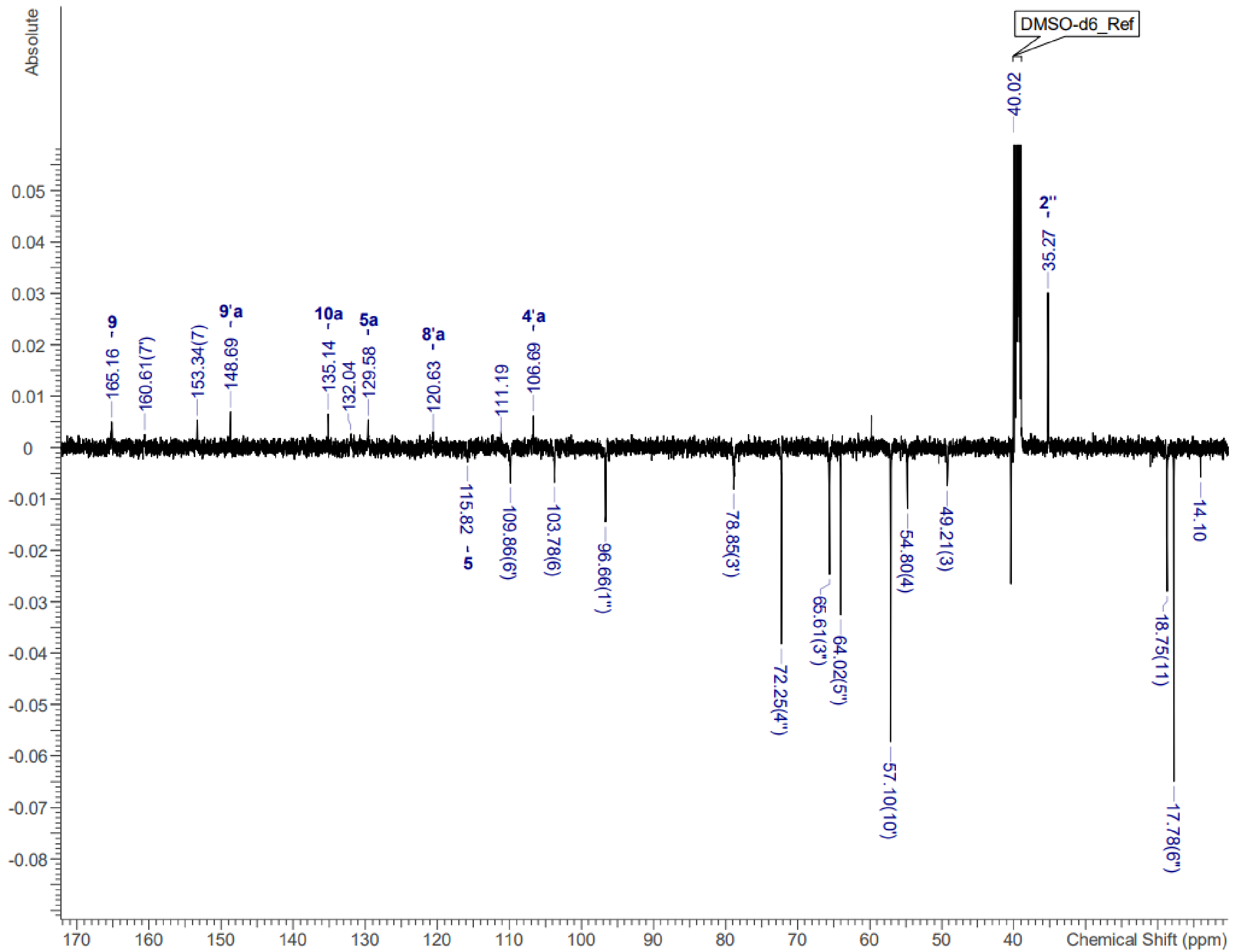

Figure S7: ^{13}C-DEPTq NMR spectrum of ruskamycin. The spectrum was recorded in DMSO-d_6 at 125 MHz.

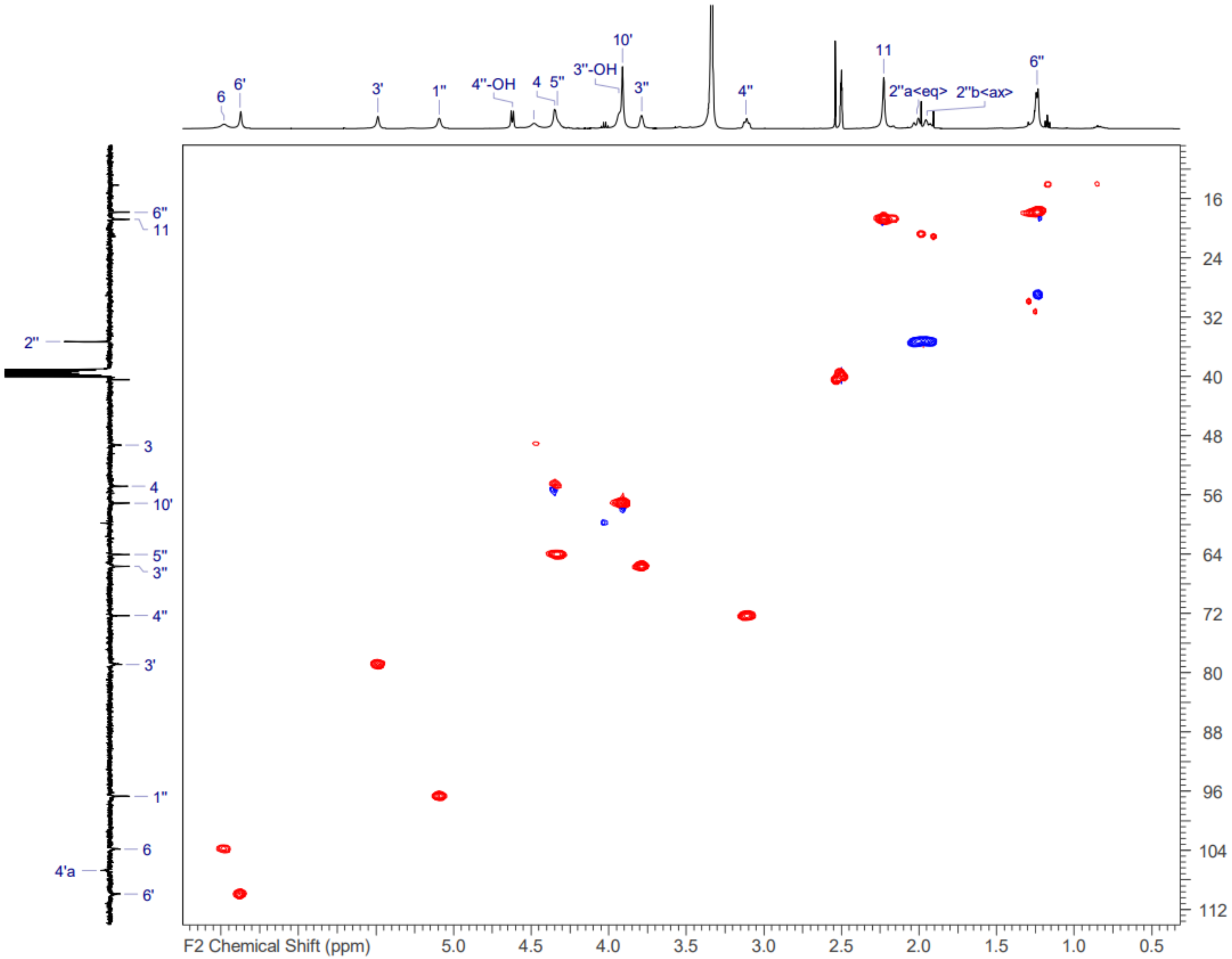

Figure S8: HSQC-DEPT spectrum of ruskamycin (DMSO-d$_6$, 500 MHz).

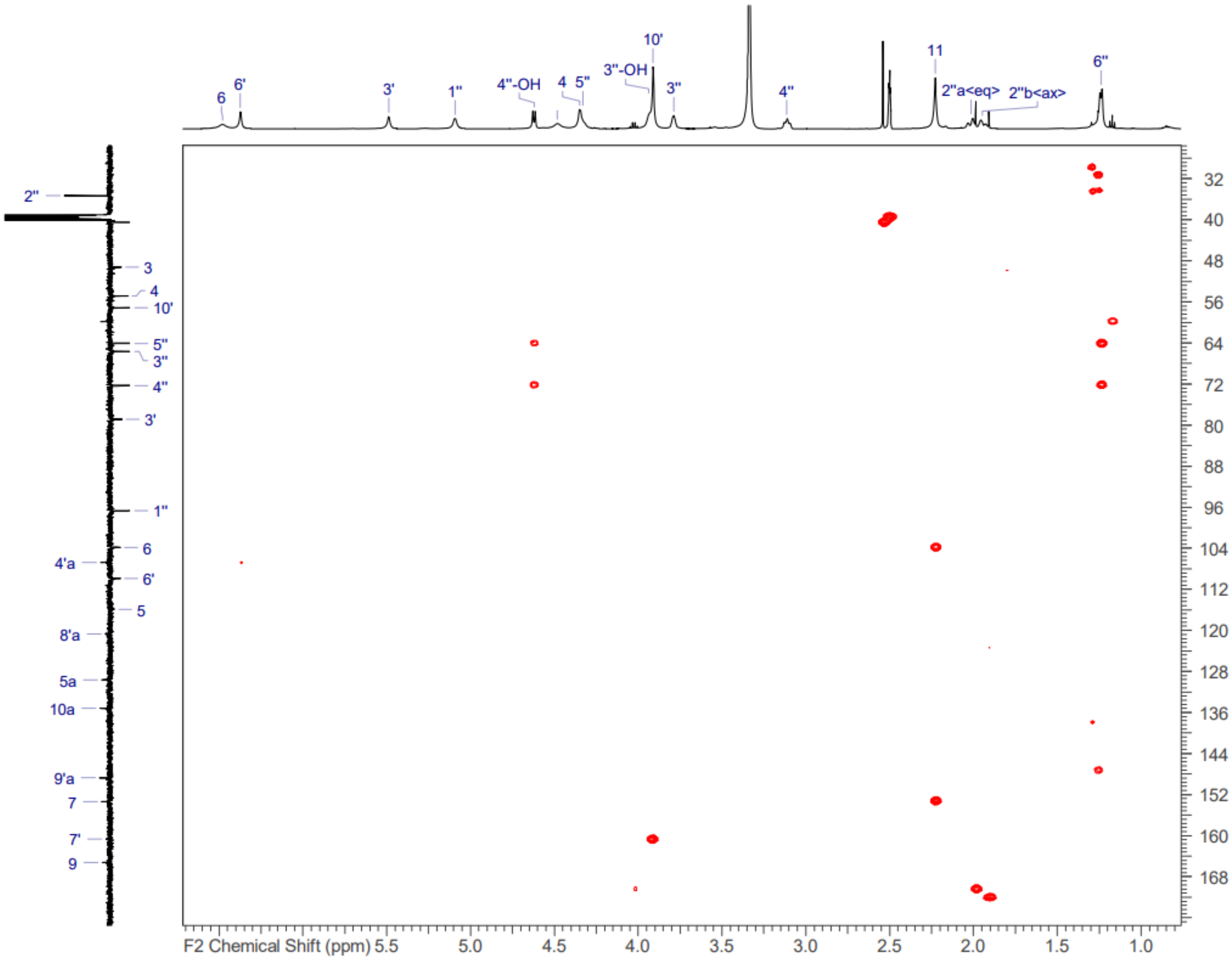

Figure S9: HMBC spectrum of ruskamycin (DMSO-d$_6$, 500 MHz).

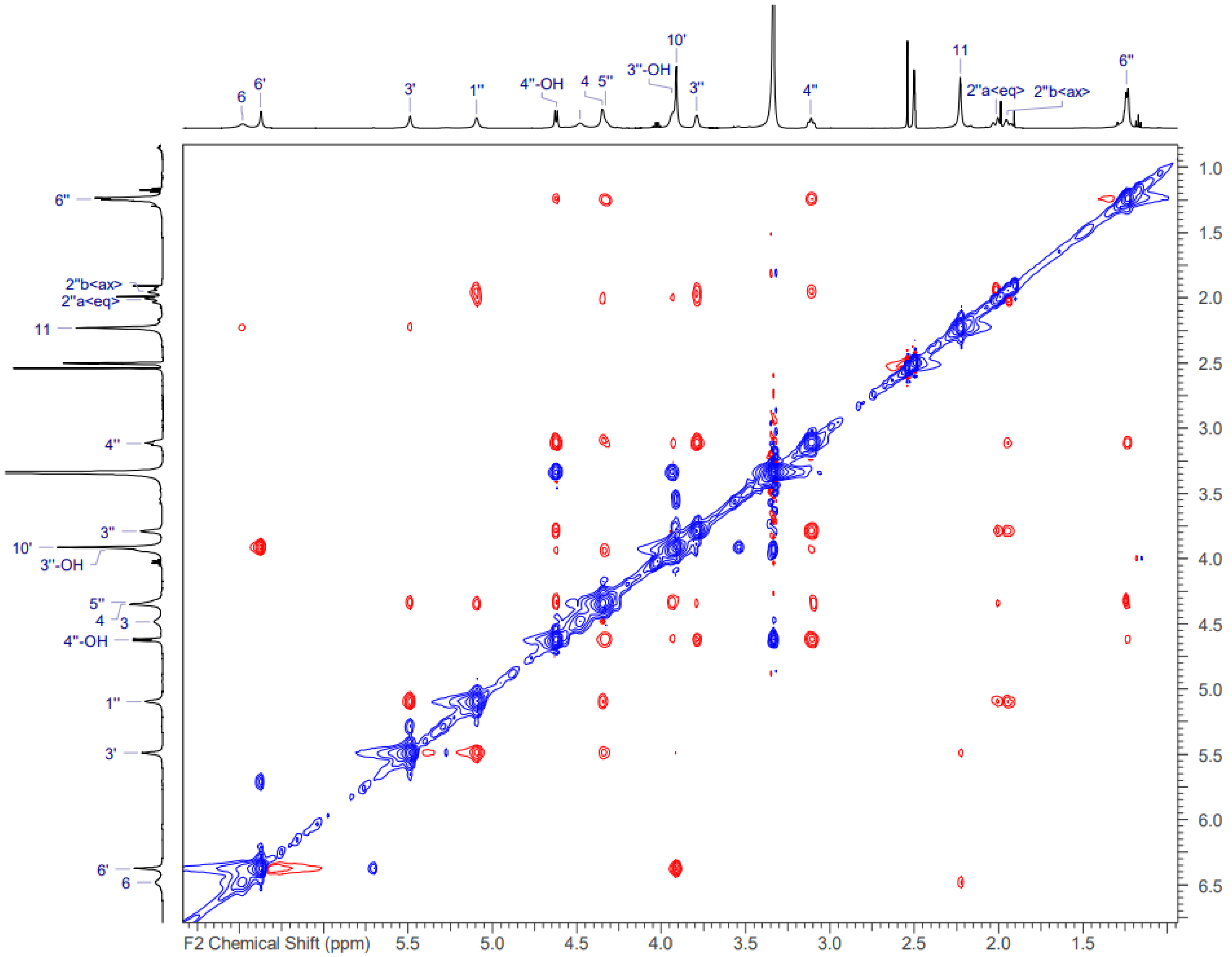

Figure S10: ROESY spectrum of ruskamycin (DMSO-d6, 500 MHz).

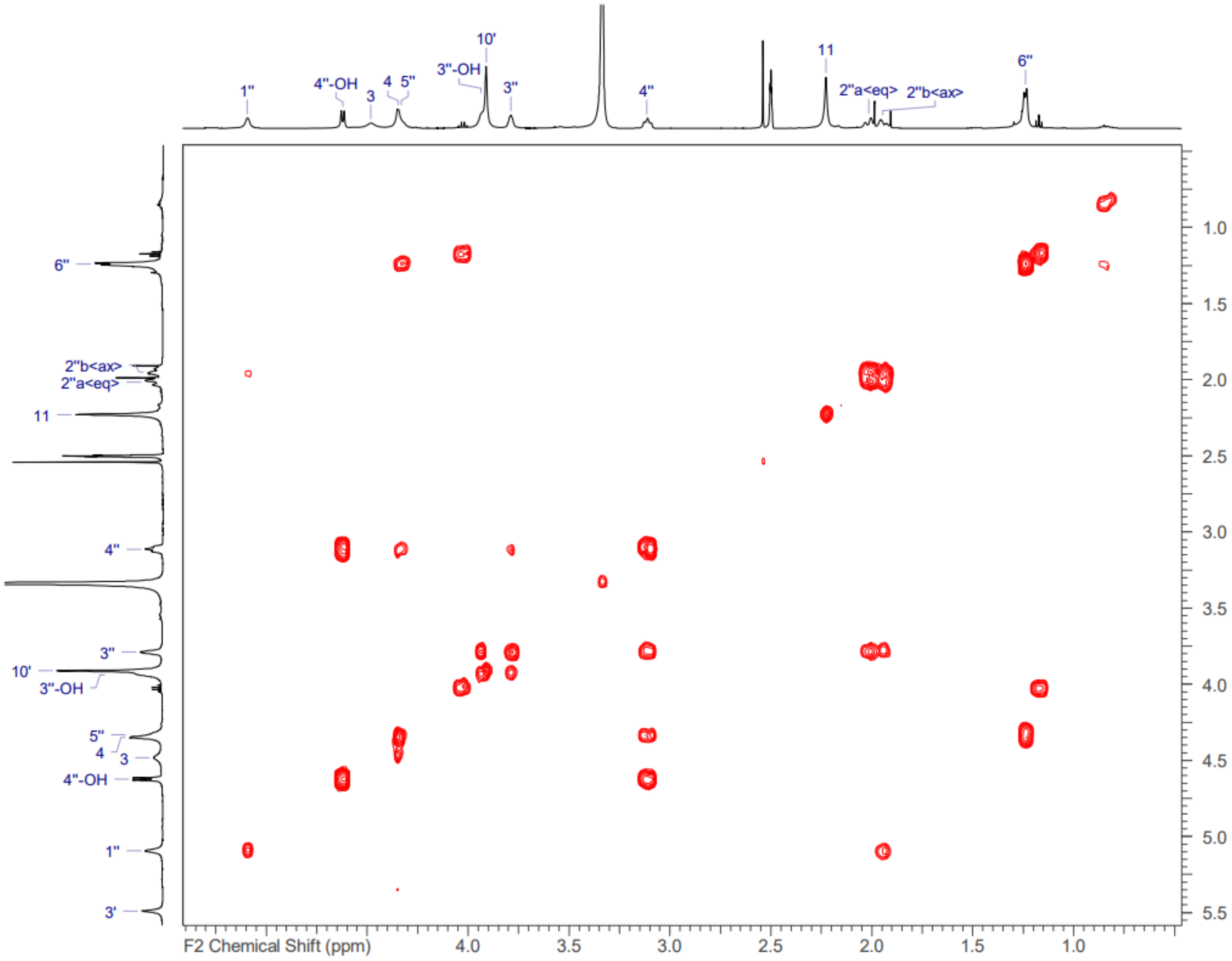

Figure S11: COSY spectrum of ruskamycin (DMSO-d6, 500 MHz).

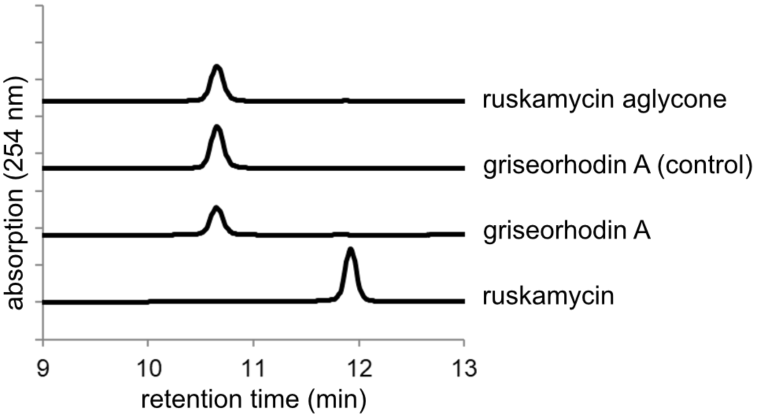

Figure S12: HPLC-DAD analysis of ruskamycin and its aglycone in comparison with griseorhodin A. The control of griseorhodin A was treated under the same conditions as ruskamycin during hydrolysis.
